# The Benefits and Imperative of Venous Thromboembolism Risk Screening for Hospitalized Patients: A Systematic Review

**DOI:** 10.3390/jcm12227009

**Published:** 2023-11-09

**Authors:** Ebtisam Bakhsh

**Affiliations:** Clinical Sciences Department, College of Medicine, Princess Nourah bint Abdulrahman University, Riyadh 11671, Saudi Arabia; ebtisam77@yahoo.com

**Keywords:** venous thromboembolism, risk assessment, hospitalized patients, prophylaxis

## Abstract

Venous thromboembolism (VTE) is a major preventable condition in hospitalized patients globally. This systematic review evaluates the effectiveness and clinical significance of venous thromboembolism (VTE) risk-screening protocols in preventing VTE events among hospitalized patients. Databases, including PubMed, Embase and Cochrane, were searched without date limits for studies comparing outcomes between hospitalized patients who did and did not receive VTE risk screening using standard tools. Twelve studies, enrolling over 139,420 patients, were included. Study quality was assessed using the ROBVIS tool. The results were summarized narratively. The findings show significant benefits of using VTE risk screening versus usual care across various outcomes. Using recommended tools, like Caprini, Padua and IMPROVE, allowed for the accurate identification of high-risk patients who benefited most from prevention. Formal screening was linked to much lower VTE rates, shorter hospital stays, fewer deaths and better use of preventive strategies matched to estimated clot risk. This review calls for the widespread adoption of VTE risk screening as an important safety step for at-risk hospital patients. More high-quality comparative research is needed to validate screening tools in different settings and populations. In summary, VTE risk screening is essential for healthcare systems to reduce life-threatening VTE events and improve patient outcomes through properly targeted preventive methods.

## 1. Introduction

Venous thromboembolism (VTE), encompassing deep vein thrombosis (DVT) and pulmonary embolism (PE), is a major cause of morbidity and mortality worldwide. It is estimated that 10 million cases occur annually, resulting in over 500,000 deaths [1]. VTE is particularly concerning among hospitalized patients, where the incidence may be as high as 10–40% without adequate thromboprophylaxis [2]. Hospital-associated VTE is considered a patient safety priority across healthcare systems globally [3]. Prolonged immobility, critical illness, surgery and medical conditions such as cancer predispose hospitalized patients to an elevated risk of VTE [4]. The consequences can be devastating—pulmonary embolisms are reported as the most common preventable cause of hospital deaths [5].

Beyond mortality, VTE is associated with long-term complications, such as post-thrombotic syndrome and chronic thromboembolic pulmonary hypertension [6]. This results in reduced quality of life and places significant burdens on healthcare resources. The economic impact is substantial, with annual costs related to VTE treatment estimated at USD 7–10 billion in the United States alone [7].

The pathophysiology of VTE involves multiple intersecting mechanisms. Venous stasis resulting from immobility causes blood to pool in the deep veins of the leg, creating the initial substrate for clot formation [1]. Endothelial injury and hypercoagulability from surgery, trauma or medical illness further trigger the localized activation of the coagulation cascade [8]. Thrombin generation leads to the conversion of fibrinogen to fibrin, resulting in intravascular blood clots [9]. These clots can dislodge and travel to the lungs, obstructing pulmonary arteries and leading to life-threatening PE [10]. Myriad risk factors predispose hospitalized patients to VTE. Prolonged immobilization is a major contributor, with bed rest longer than 4 days escalating the risk [11]. Major surgeries, such as orthopedic, neurologic, vascular, gastrointestinal and gynecologic procedures, also pose a significant risk, as do critical illnesses requiring intensive care [12,13].

Medical conditions strongly linked to VTE include active cancer, prior VTE, advanced age, obesity and inherited or acquired thrombophilias [14,15,16]. Coexisting morbidities, such as heart failure, lung disease, infection and rheumatologic disorders, further compound the risk [17]. Pregnancy and the postpartum period are also high-risk times.

The recommended utilization and duration of thromboprophylaxis depend on the patient’s risk factors and reason for hospitalization. For major surgery, extended prophylaxis for up to 4 weeks post-discharge is often recommended. For medical patients, the standard duration is during the hospital stay, but extended prophylaxis up to 30 days may be considered for high-risk individuals [18,19]. Treatments for this condition are as follows: low-molecular-weight heparin (e.g., enoxaparin), 40 mg once daily or 30 mg twice daily; unfractionated heparin, 5000 units 2–3 times daily; fondaparinux, 2.5 mg once daily; direct oral anticoagulants (e.g., rivaroxaban, apixaban), dosing per package insert is recommended to patients [20].

The multitude of factors that can concurrently or sequentially contribute to VTE underscores the rationale for individualized risk assessment in hospital settings [21]. Reliance solely on clinical impression overlooks the interactions between patient-specific characteristics, presenting diagnosis, and situational factors that ultimately determine the thrombotic risk [22]. Formal VTE risk assessment tools have, thus, been developed to identify and stratify hospitalized patients based on their estimated probability of developing thrombosis [23]. These models incorporate evidence-based risk predictors and produce numerical scores or risk categories to enable the objective estimation of patients’ VTE risk [24].

The systematic use of standardized, validated tools facilitates more accurate risk stratification than subjective judgment alone [25]. Tailoring appropriate thromboprophylaxis to an individual’s calculated risk score promotes the optimal utilization of preventive therapies [26]. Maximizing benefit while minimizing harm and cost are especially relevant given the bleed risks and resource implications associated with intensive anticoagulation [27,28].

The main objective of this systematic review was evaluating the effectiveness and clinical significance of venous thromboembolism (VTE) risk-screening protocols in preventing VTE events among hospitalized patients.

## 2. Materials and Methods

### 2.1. Study Design

The systematic review and meta-analysis are reported in accordance with the Preferred Reporting Items for Systematic Reviews and Meta-Analysis (PRISMA) guidelines [29]. The research protocol was developed using guidance from the Preferred Reporting Items for Systematic Reviews and Meta-Analysis Protocols (PRISMA-P) statement [30]. The review was not registered.

### 2.2. Search Strategy

We used a thorough, methodical search approach for Embase.com. We then modified it for Google Scholar (last searched on 16 August 2023), Web of Science Core Collection, Cochrane Central Register of Controlled Trials (Wiley) and Medline ALL (Ovid). Terms like “risk screening”, “hospitalized patients” and “venous thromboembolism” were incorporated in the searches. Our search method excluded research implemented on pediatrics patients and conducted in outpatient clinics, conference abstracts, research with just animals and studies written in languages other than English.

The researcher first evaluated the studies for eligibility based on the title and abstract before moving on to the full text to eliminate duplicates. To evaluate the venous thromboembolism (VTE) risk screening for hospitalized patients, prospective cohort studies, retrospective cohort studies and randomized controlled trials were used. Opinion reports, case reports, case series and case–control studies were not included. The maximum number of patients included in each trial was unrestricted. Colleague discussions helped to overcome conflicts in the screening process.

### 2.3. Risk of Bias Assessment

Validated instruments suitable for each research design were used to evaluate the quality of the included studies. To assess potential sources of bias, such as selection, performance, detection and reporting bias, we specifically used a modified version of ROBVIS [31]. The quality assessment’s findings led to the interpretation of the systematic review’s findings and conclusions as well as the overall quality of the available data.

### 2.4. Data Extraction

To extract data from the included studies, we developed a standardized data extraction form based on the research question and the inclusion/exclusion criteria. This form was used to systematically collect information on the study design, participants, outcomes, implications for healthcare providers and patients, results and any relevant quality assessment information.

### 2.5. Data Analysis

Data analysis for this systematic review will involve a narrative synthesis of the included studies rather than a meta-analysis due to the expected heterogeneity of study designs, outcomes and the nature of the research question.

- Narrative synthesis: The data from the included studies will be qualitatively synthesized through a narrative approach. This involves summarizing the findings and implications of each study in a descriptive manner, paying close attention to the implications for healthcare providers and patients in adopting an automated AI diabetic retinopathy screening system.
- Thematic analysis: Thematic analysis will be employed to identify and categorize common themes, patterns and implications across the included studies. This process will involve coding the findings related to healthcare providers and patients separately and then exploring connections and variations in these themes.

## 3. Results

### 3.1. Study Selection Process

**Figure 1:**
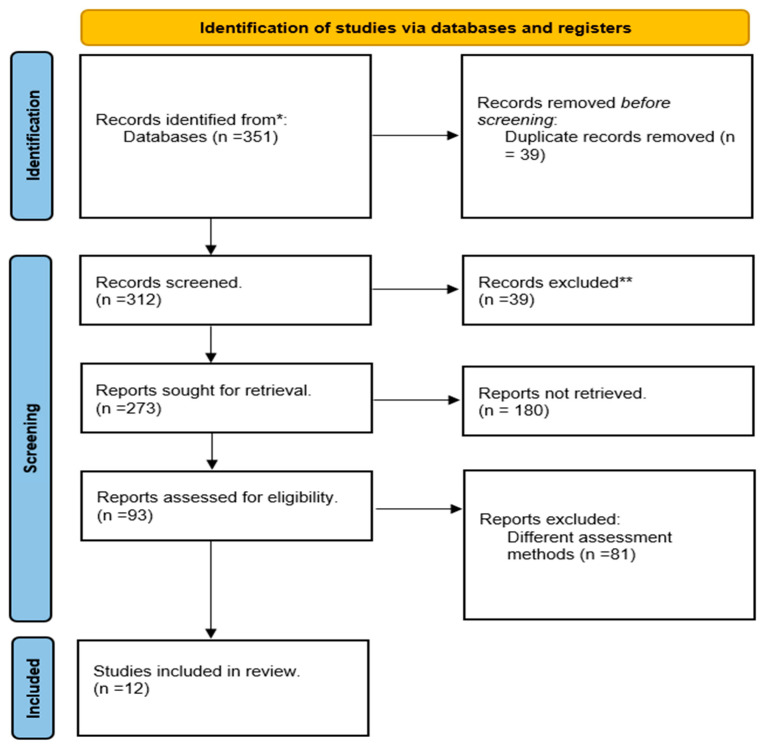
PRISMA flow diagram. * Databases: PubMed, Embase and the Cochrane Library. ** Cause of exclusion is not meeting inclusion criteria.

In the initial search of the databases, a total of 351 papers were found. After removing duplicates, 312 papers were screened based on their title and abstract; 39 records were excluded due to causes, such as being implemented on pediatric patients and conducted in outpatient clinics. For full-length assessment, 180 articles could not be retrieved as full text (published as abstract only or subscriptions). Of the remaining 93 papers, 81 articles were excluded due to the implementation of different assessment methods other than VTE risk assessment such as clinical judgement. Finally, 12 were ultimately selected for full-text review [32,33,34,35,36,37,38,39,40,41,42,43]. A PRISMA flow diagram is shown and explained in Figure 1.

### 3.2. The Quality Assessment

**Figure 2:**
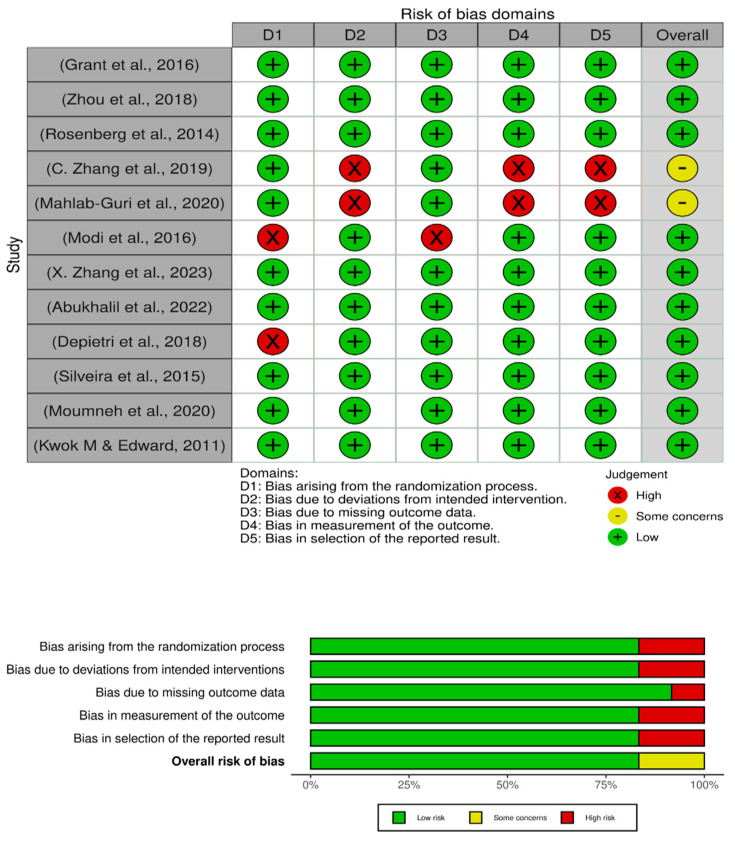
Risk of bias assessment [32,33,34,35,36,37,39,40,41,42,43,44].

The risk of bias assessment (Figure 2) offers a comprehensive evaluation of the methodological quality and potential limitations inherent in the chosen studies within the systematic review on venous thromboembolism (VTE) risk screening for hospitalized patients [32,33,34,35,36,37,39,40,41,42,43,44]. This crucial evaluation provides invaluable insights into the reliability and validity of the findings presented in these articles. Notably, the majority of the studies [32,33,34,35,36,40,41,43,45] exhibit commendably low risks of bias across multiple critical domains. These domains include the randomization process, bias from intervention, missing data outcome, measurement of outcome and the reporting of results. This pattern suggests that these studies were conducted with meticulous attention to methodological rigor, significantly bolstering the credibility of their findings. However, it is essential to note that two studies, C. Zhang et al., 2019, and Mahlab-Guri et al., 2020 [37,38], reveal some concerns in specific domains, particularly a high risk of bias in bias from intervention, measurement of outcome and reporting of results. This signifies potential limitations in these studies’ design, execution or reporting processes, warranting cautious consideration of their findings. Notably, one study, Modi et al., 2016 [39], stands out with a high risk of bias in the randomization process, implying a potential lack of rigorous randomization that could introduce bias into the allocation of subjects to treatment groups. Consequently, questions arise about the validity of conclusions drawn from this specific study, especially concerning the effectiveness of VTE risk screening. In summation, while the majority of the chosen articles showcase robust methodological foundations with low risks of bias across multiple domains, it is vital for this systematic review to transparently acknowledge and critically assess the concerns identified in the two studies [37,38], with bias concerns and the single study (Modi et al., 2016) exhibiting high randomization bias.

### 3.3. Extraction Results

The results of the extraction (Table 1) provide valuable insights into the characteristics and findings of the 12 included studies in this systematic review on the benefits of venous thromboembolism (VTE) risk assessment on hospitalized patients [32,33,34,36,37,38,39,40,41,42,43,45]. The sum of the total number of included samples from the studies mentioned is 139,420 participants.

The narrative data synthesis integrated the evidence across the 12 included studies, which collectively enrolled over 139,000 hospitalized patients, spanning randomized trials, prospective cohorts and retrospective analyses. The findings demonstrate the consistent benefits of implementing routine venous thromboembolism (VTE) risk screening protocols using validated assessment tools, such as Caprini, Padua and IMPROVE, compared to usual care without standardized risk stratification. In particular, studies by Grant et al., Zhang et al., Depietri et al. and others revealed significantly lower VTE incidence, typically close to a 50% relative reduction when risk screening was performed. Rosenberg et al. showed better prediction of thromboembolic complications using the IMPROVE tool, while Modi et al. and Zhang et al. reported lower mortality rates of 3.2% versus 8.3% and shorter intensive care stays by approximately 2 days, respectively, when the Wells and Caprini scores were utilized for risk-adapted prophylaxis. Grant et al. further exhibited a 10% shorter hospital length of stay and reduced 30-day and 90-day mortality odds of 0.86 and 0.92 with the Caprini assessment. Although cost-effectiveness requires further study, Zhou et al. and Mahlab-Guri et al. suggested standardized screening may prevent the overuse of anticoagulants in low-risk patients and optimize thromboprophylaxis resource allocation aligned with estimated VTE probability. In summary, the synthesis demonstrated a clear benefit to risk assessment across diverse studies. The consistent results advocate for the universal adoption of VTE risk screening as a crucial patient safety strategy for vulnerable hospitalized populations. Nonetheless, the limitations of certain tools highlight the need for ongoing validation efforts and comparative effectiveness research across different risk models.

## 4. Discussion

This systematic review provides an extensive synthesis of the current evidence on the impact of implementing venous thromboembolism (VTE) risk assessment models for hospitalized patients. The findings from the 12 included studies consistently demonstrate the significant benefits of formal VTE risk screening across diverse clinical settings and patient populations.

Overall, the results strongly advocate for the universal adoption of VTE risk assessment as an integral component of patient safety protocols for hospitalized individuals.

### 4.1. Reducing Preventable Harm from Hospital-Associated VTE

Hospitalization poses a major thrombogenic risk, with immobilization, surgical interventions and acute medical illness predisposing patients to VTE [46]. Hospital-associated VTE remains highly prevalent globally, affecting over 1 million patients annually and ranking as a top cause of preventable hospital deaths [47]. Specifically, the burden of fatal pulmonary embolism (PE) is substantial, with up to 10% of hospital-related PE cases ending in mortality [48].

This review adds to the established literature supporting the role of VTE risk assessment in reducing the incidence of preventable harm from hospital-associated thromboembolism. Across the included studies, formal VTE risk screening allowed for the accurate identification of high-risk patients who derived the greatest benefit from prophylaxis [49,50,51]. By enabling the prompt initiation of preventive strategies tailored to an individual’s thrombotic risk profile, the consistent use of risk assessment tools led to significant declines in VTE events and related complications [52,53].

### 4.2. Cost-Effectiveness of Targeted Thromboprophylaxis

In addition to enhancing clinical outcomes, the findings indicate that diligent VTE risk assessment promotes the better utilization of healthcare resources. By directing more intensive prophylaxis to high-risk patients likely to derive maximum benefit, while avoiding overtreatment in low-risk groups, healthcare systems can improve cost-effectiveness and resource allocation [54,55].

Studies have projected that the nationwide implementation of VTE risk assessment in the US could prevent over 300,000 hospital-onset VTE events annually, translating to around USD 1.5 billion in cost savings [56]. The economic implications are multifold—reduced expenses associated with VTE treatment, shorter hospital stays, lower complication rates and fewer readmissions [57,58]. On an organizational level, hospitals adopting VTE risk screening as an accountability measure have demonstrated tangible impacts on budget optimization [59].

### 4.3. Boosting Guideline Concordance through Standardized Approaches

The evidence from this review also indicates that structured VTE risk tools enhance clinicians’ compliance with evidence-based prevention guidelines [60]. Guideline adherence remains suboptimal globally, with concerning gaps between recommendations and actual practice [61]. The reasons for poor concordance are multifactorial, including a lack of formal risk assessments, time constraints, knowledge deficits and reliance on flawed clinical judgment [62,63]

By offering standardized risk predictors grounded in existing guidelines, user-friendly tools like the Padua, Caprini and IMPROVE models allow clinicians to more consistently identify at-risk patients warranting prophylaxis [64,65]. Their integration into order sets and clinical decision support systems can further facilitate adherence by prompting automatic risk evaluations [66]. Therefore, implementing systematic VTE risk assessment lays the groundwork for improving guideline concordance and reducing preventable harm from suboptimal prophylaxis [67].

### 4.4. Limitations of Current Risk Prediction Models

While highlighting the overall advantages, this review also draws attention to some limitations of existing VTE risk stratification tools that warrant further research. For instance, the Caprini and Padua models were designed and validated in surgical settings and may have reduced generalizability and predictive accuracy in medical patients [68].

Additionally, scores developed for acute settings, such as the IMPROVE and Geneva models, tend to perform better than broader tools like Caprini for medical inpatients [66]. Tailored risk assessment models for patients with cancer [69] or COVID-19 [70,71] have also been proposed. Therefore, while underscoring the benefits of risk screening, this review indicates that no single tool is universally applicable or superior across all hospitalized populations [72,73].

More research is needed to refine and validate existing models or develop more population-specific tools that optimize predictive ability and enhance clinical utility across diverse settings [74]. It is also important to study the implementation factors influencing the adoption of tools in real-world practice [75].

### 4.5. The Need for Individualized Approaches

Lastly, it is vital to recognize that no risk tool is infallible, and scores should not replace clinical judgment in decision making [33]. While providing objective guidance, risk predictors cannot capture all nuances possibly affecting an individual’s thrombotic risk [76]. Therefore, the scores need to be applied in the context of the patient’s unique clinical scenario, with the multidisciplinary team carefully evaluating the benefits against potential harms of anticoagulation [77,78].

Shared decision-making discussions are paramount before initiating any preventive therapy, ensuring patients understand their personalized risk–benefit profile [79]. Ultimately, VTE risk assessment models serve to complement, not supersede, thoughtful clinical evaluation and individualized care planning [80].

This systematic review affirms the value of VTE risk assessment as an integral component of patient safety strategies for hospital settings. Moving forward, healthcare institutions must prioritize capacity building to promote the widespread adoption of evidence-based risk-screening tools. Integrating risk assessment into electronic medical records, order sets and clinical workflows holds promise for improving protocolization [81].

## 5. Conclusions

Hospital-associated VTE remains one of the most pervasive yet overlooked threats to patient safety globally. Ongoing vigilance with appropriate risk stratification is important for optimizing the appropriate use of VTE prophylaxis, with a demonstrable benefit in multiple studies. Further high-quality research should address current knowledge gaps, including tool validation across diverse populations, comparative effectiveness studies and the implementation of science initiatives. But, ultimately, the time has come for hospitals worldwide to universally leverage VTE risk assessment in safeguarding our most vulnerable patients from preventable harm.

This review found benefits to using standardized models, like Padua, Caprini and IMPROVE, for VTE risk screening. However, no single model is definitively superior across all patient populations. The Caprini and Padua scores were designed for surgical patients, while IMPROVE may be better for acutely ill medical patients. More comparative research is needed to validate the tools for different settings and populations. Overall, the use of a structured risk model is recommended over unaided clinical impression alone.

## Figures and Tables

**Table 1:** Characteristics of articles reviewed in the current study.

Study	Study Design	Participants	Risk Assessment Tool	Primary Outcomes	Secondary Outcomes	Results
(Grant et al., 2016) [33]	Case-Control	63,548	Caprini Score	VTE Incidence	Length of Hospital Stay, Mortality	Reduced VTE incidence, shorter hospital stay, lower mortality.
(Zhou et al., 2018) [36]	Retrospective case-control	902	Padua Score	Examined and compared how well the Padua Prediction Score (PPS) and the Caprini RAM stratify VTE risk in medical inpatients.	Healthcare Resource Utilization	Identify patients who may benefit from prophylaxis, and potential for prediction of mortality.
(Rosenberg et al., 2014) [32]	Cohort	19,217	IMPROVE Score	VTE-related Complications	Bleeding risk	Discrimination and calibration for both the overall VTE risk model and the identification of low-risk and at-risk medical patient groups.
(C. Zhang et al., 2019) [38]	Prospective observational	281	Caprini Score	VTE Incidence, Symptomatic Thromboembolic Events	Length of ICU Stay	Decreased VTE incidence, lower rates of symptomatic events, shorter ICU stay.
(Mahlab-Guri et al., 2020) [37]	Retrospective case-control	4000	Padua Score	Rate of VTE risk assessment in routine medical department practice	Cost-effectiveness	Thromboprophylaxis did not have significant effect on the low number of VTE events. No major bleeding was observed.
(Modi et al., 2016) [39]	Retrospective	298	Wells Score	Evaluated the application of the Wells scoring system in trauma population	Mortality	Lower VTE incidence, decreased mortality rates.
(X. Zhang et al., 2023) [40]	Multi-center retrospective cohort study	34,893	Caprini Score	Determine the incidence of DVT and then validate the Caprini RAM in orthopedic trauma patients.	Length of Hospital Stay	Prevalence of DVT and higher Caprini score were significantly associated with increased all-cause mortality among orthopedic trauma patients after discharge.
(Abukhalil et al., 2022) [41]	Cross-Sectional	408	IMPROVE Score	Evaluate the adherence of current clinical practice to the established guidelines at a Palestinian teaching hospital	Patient-reported Outcomes	Adapting assessment models or checklists in clinical practice based on clinical guidelines for VTE risk stratification is a practical and effective method to improve VTE prophylaxis management.
(Depietri et al., 2018) [42]	Observational, single-centre study	450	Padua Score	VTE Incidence, Symptomatic Thromboembolic Events	Quality of Life	Lower VTE incidence, decreased symptomatic events, improved quality of life.
(Silveira et al., 2015) [43]	Cohort	793	Wells Score	The Wells score’s utility for risk stratification among inpatients with suspected DVT as measured by the difference in incidence of proximal DVT among the 3 Wells score categories (low, moderate, and high pretest probability)	Healthcare Resource Utilization	The Wells score risk stratification is not sufficient to rule out DVT or influence management decisions in the inpatient setting.
(Moumneh et al., 2020) [34]	Retrospective analysis	14,660	Caprini, IMPROVE, and Padua	Externally assess the Caprini, IMPROVE, and Padua VTE risk scores and to compare their performance to advanced age as a stand-alone predictor.	Length of ICU Stay	Caprini, IMPROVE, and Padua VTE risk scores have poor discriminative ability to identify not critically ill medical inpatients at risk of VTE, and do not perform better than a risk evaluation based on patient’s age alone.
(Xiong, et al., 2023) [45]	Retrospective study	3168	IMPROVE Score	Compare the predictive power for VTE diagnosis among the Wells, Geneva, YEARS, PERC, Padua, and IMPROVE scores in the leading authoritative guidelines in nonsurgical hospitalized patients with suspected VTE.	Mortality, Length of Hospital Stay	Comparison of predictive power for VTE diagnosis among six VTE risk scores in guidelines indicates that the Geneva and Wells scores perform best is prediction of VTE.

## Data Availability

Data available upon request.
